# Multivariate patterns of brain-behavior associations across the adult lifespan

**DOI:** 10.18632/aging.203815

**Published:** 2022-01-10

**Authors:** Gaelle E. Doucet, Noah Hamlin, Anna West, Jordanna A. Kruse, Dominik A. Moser, Tony W. Wilson

**Affiliations:** 1 Institute for Human Neuroscience, Boys Town National Research Hospital, Omaha, NE 68010, USA; 2 Institute of Psychology, University of Bern, Bern, Switzerland; 3 Child and Adolescent Psychiatry, University Hospital Lausanne, Lausanne, Switzerland; 4 Department of Pharmacology and Neuroscience, Creighton University School of Medicine, Omaha, NE 68178, USA

**Keywords:** healthy aging, higher-order cognition, multivariate analyses, MRI, brain networks

## Abstract

The nature of brain-behavior covariations with increasing age is poorly understood. In the current study, we used a multivariate approach to investigate the covariation between behavioral-health variables and brain features across adulthood. We recruited healthy adults aged 20–73 years-old (29 younger, mean age = 25.6 years; 30 older, mean age = 62.5 years), and collected structural and functional MRI (s/fMRI) during a resting-state and three tasks. From the sMRI, we extracted cortical thickness and subcortical volumes; from the fMRI, we extracted activation peaks and functional network connectivity (FNC) for each task. We conducted canonical correlation analyses between behavioral-health variables and the sMRI, or the fMRI variables, across all participants. We found significant covariations for both types of neuroimaging phenotypes (*ps* = 0.0004) across all individuals, with cognitive capacity and age being the largest opposite contributors. We further identified different variables contributing to the models across phenotypes and age groups. Particularly, we found behavior was associated with different neuroimaging patterns between the younger and older groups. Higher cognitive capacity was supported by activation and FNC within the executive networks in the younger adults, while it was supported by the visual networks’ FNC in the older adults. This study highlights how the brain-behavior covariations vary across adulthood and provides further support that cognitive performance relies on regional recruitment that differs between older and younger individuals.

## INTRODUCTION

Brain organization throughout the lifespan has been associated with multiple genetic, molecular, behavioral, and environmental factors [1–6]. Large-scale studies have begun to address the complexity of the brain-behavior-environment associations. In the Human Connectome Project -Young Adult (HCP-YA), a U.S. study done on healthy adults aged 22–37 years, personal attributes and environmental factors were correlated with brain structure and functional connectivity following a positive-negative axis [7, 8]. In other words, the majority of behavioral variables that correlated positively with more efficient brain patterns (e.g., stronger network integration) were positive subject traits and measures (such as education, income, IQ, life-satisfaction), while those that correlated negatively were mostly negative subject measures (such as body-mass index (BMI), smoking, age). This finding has been consistently reproduced using independent samples of young adults [9], youth [10–12] and clinical samples [9, 13–16].

Throughout adulthood, age has been robustly established as one of the strongest negative contributors to brain function using multivariate [7, 8] and univariate [17–20] approaches. Healthy aging has been associated with regional brain atrophy [17, 21, 22], reduced brain network integrity [20, 23–25] and changes in regional connectivity [20, 24–26]. Beyond this negative impact, a study by Miller et al. (2016) [17] that involved 5,000 participants aged 44–78 years old suggested that aging alters the brain-behavior-environment associations in two ways. First, these associations may vary by age range (i.e., early versus middle versus late adulthood). Second, within older adults, age-related cognitive decline seems to be a major source of variability for these associations, suggesting that age-related impact varies by neural parameter and cognitive function investigated [17]. However, there is a limited number of neuroimaging studies that focused on multivariate associations in late adulthood or across the adult lifespan, even though a key goal of cognitive neuroscience and multiple federal funding initiatives is to characterize how age-related variation in brain organization relates to cognitive, psychosocial and environmental factors, as this may help identify the relevant brain mechanisms that signify the transition from healthy aging to neurodegenerative states [27, 28].

In this context, the aim of the present study was to further characterize the impact of healthy aging on patterns of multivariate covariation between non-imaging and imaging variables and identify the most salient features that drive these associations in older relative to younger healthy adults. To achieve this aim, we recruited 59 healthy adults (29 adults aged 19–32 years-old and 30 adults aged 55–72 years-old). We collected a high-resolution structural scan, three cognitive functional MRI (fMRI) tasks that related to working memory, visual episodic memory and language function, respectively, and a resting-state fMRI scan in one session. These tasks assess three of the most critical components of cognitive processing, underlying many more complex functions. Using the structural scan, we extracted structural morphometric measures (subcortical volumes and cortical thickness), while with the fMRI data we extracted individual measures of task-related and resting-state functional network connectivity (FNC) and task-related brain activation. We extracted both types of brain functional features because they: (a) are among the most commonly analyzed measures in fMRI, (b) have been suggested to reflect different brain mechanisms [29, 30] and (c) are related to distinct behavioral and cognitive features [9, 17]. Lastly, in aging models, it appears that functional connectivity versus task-based activation techniques may reveal different aspects of recruitment of additional regions to support and/or compensate cognitive processing [31–34]. Overall, we aimed to: (1) test the relative sensitivity of these brain features (structural and functional) to maintain optimal cognitive function and health, and (2) compare these associations between early and late adulthood. In this context, we ascertained non-imaging/health factors that related to medical history, metabolism, cognition, lifestyle factors and physical health (*n* = 59 variables). These variables were selected based on previous fMRI studies which demonstrated their relevance in understanding the covariation linking brain activity, demographics, and behavior [7, 8, 17]. Using these multimodal data, we conducted sparse canonical correlation analyses (sCCA) [35, 36] to investigate the underlying relationship between the neuroimaging and health/demographic/lifestyle factors in the whole sample, and further to identify the differential weights of contribution of the top variables in each model in the younger versus older adults. We used sCCA because this multivariate method is ideally suited for predicting one dataset from the other while accounting for correlations between variables [36, 37]. CCA is a powerful multivariate tool to simultaneously investigate relationships among multiple variables and/or datasets, and further enables one to separate distinct biological processes with opposing relationships between variables [17, 36]. In particular, we chose to conduct sCCA because it is tailored to the analyses of high-dimensional datasets in which variables are expected to be correlated and does not require data reduction. It has the further advantage over classic CCA that more observations than participants is acceptable and produces reliable results even in smaller samples [36, 38]. Considering preexisting evidence, our working hypotheses were that beyond an overall negative effect of age at the level of the whole sample, the brain-behavior covariation would vary between the two age groups. Particularly, we expected that: (i) age would have a stronger (negative) impact on brain efficiency in the older group, and (ii) top contributors to the brain-behavior covariations would differ between the two groups. In particular, we expected that health- and metabolism-related measures (e.g., glucose rate, physical activity) would contribute more to brain integrity in older than in younger adults. We also hypothesized that cognitive variables shown as strong positive predictors of brain integrity in youth and young adults [7–9, 11] would show a lower contribution in older adults, because healthy aging has been typically associated with slight declines and slowing in general cognitive function [39, 40]. Finally, (iii) older adults would show a more widespread functional and structural regional contribution to support higher-order cognitive function when compared to the younger adults. The latter follows the compensation-related utilization of neural circuits hypothesis (CRUNCH) [40], and more recently the revised scaffolding theory of aging and cognition (STAC-r) model [34, 41]. These aging models support the overall idea that age-specific increases in regional recruitment provide support to maintain higher cognitive performance in older individuals, with this positive association enhanced by enriched lifestyle (e.g., higher physical activity). Lastly, we hypothesized that measures of structural morphometry would emerge as stronger contributors to behavior than the functional features as our previous study found in young adults [9].

## RESULTS

### Sparse CCA between non-imaging and sMRI datasets

**Figure 1:**
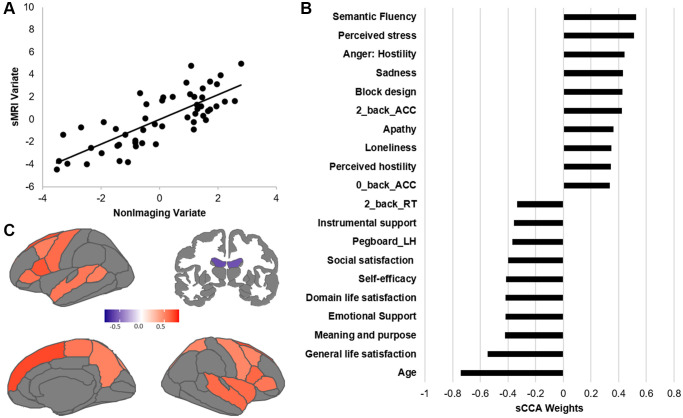
**Results of the sCCA between non-imaging and sMRI datasets across all participants.** (**A**) Significant correlation across all participants (r = 0.612, *p* = 0.0001). (**B**) Top behavioral–health variables most strongly associated with the imaging variate. (**C**) Top sMRI variables positively associated with the behavioral–health variate. Details of each variable in Supplementary Table 5. Contributions of all variables are provided in Supplementary Tables 1 and 2.

Across all participants, we found a significant association between the non-imaging dataset and the sMRI dataset (r = 0.785, *p* = 0.0004; Figure 1A). In the non-imaging dataset, the highest positive weights were attributed to measures related to intelligence and higher cognitive function (verbal fluency, block design), better accuracy during the fMRI tasks, and emotions related to negative affect and perceived negative social relationships (Figure 1B, Supplementary Table 1). In contrast, older age, emotions related to well-being, and lower motor dexterity (e.g., slower reaction times) displayed the strongest negative contribution. In the imaging dataset, the structural features with the highest positive contributions were the cortical thickness of lateral and medial frontal regions as well as superior temporal cortex (Figure 1C, Supplementary Table 2). In contrast, the top negative structural contributors were the volume of the lateral ventricles.

**Figure 2:**
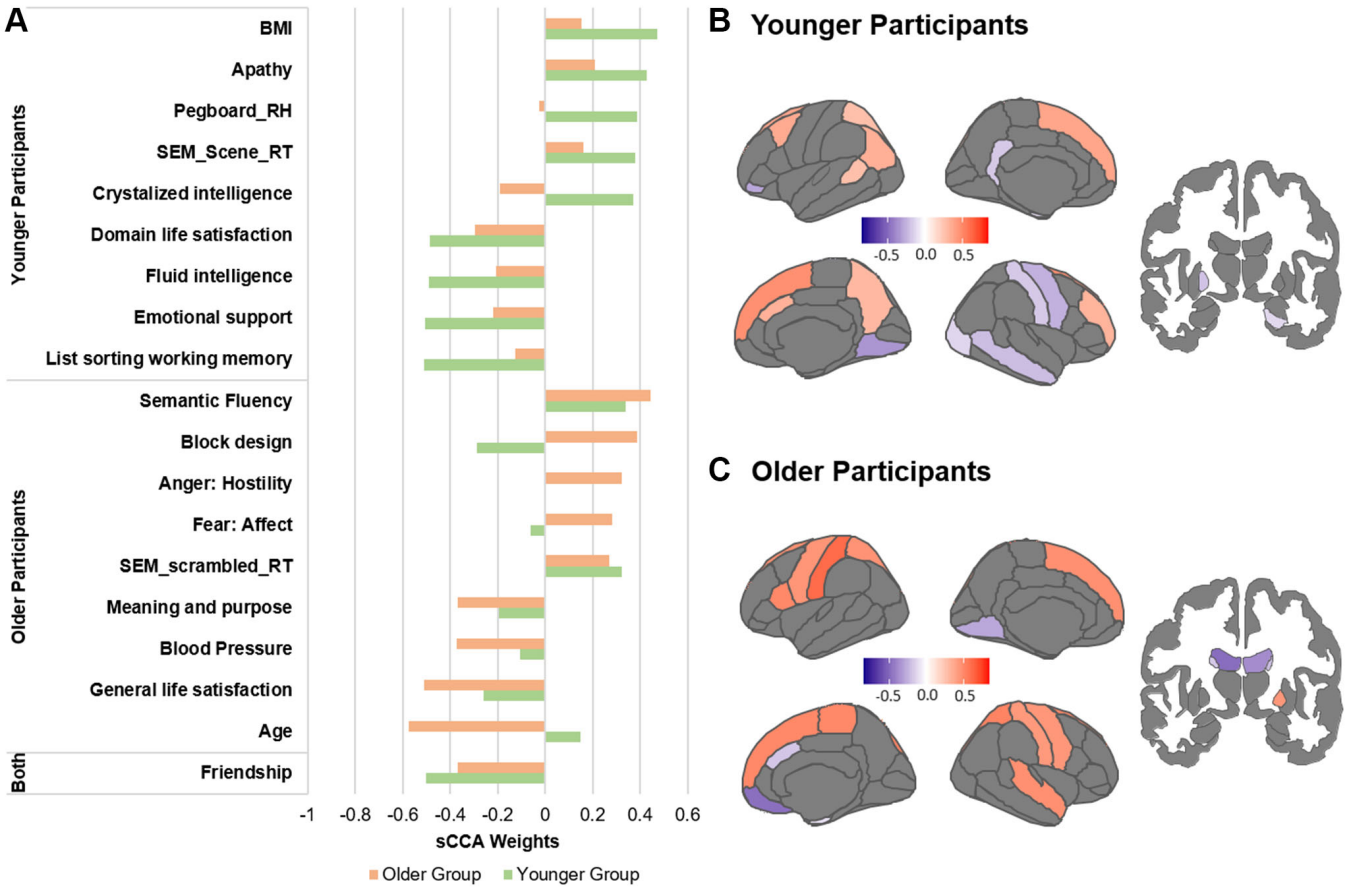
**Top contributors in the sMRI sCCA in younger and older groups, separately.** (**A**) Top behavioral–health variables most strongly associated with the imaging variate in each subgroup. (**B**) Top sMRI variables associated with the behavioral–health variate in the younger group. (**C**) Top sMRI variables associated with the behavioral–health variate in the older group. Details in Supplementary Tables 1, 2 and 5.

We further identified the top contributors to the covariation within each age group (Figure 2). While we only report and discuss the top variables, all variable contributions are provided in Supplementary Tables 1 and 2. In the non-imaging dataset, the variables with the highest correlations with the sMRI dataset were mostly different between the two groups. In the younger group, the non-imaging variables with the strongest contributions were related to higher BMI, reaction times, apathy and cognitive ability. In the imaging dataset, cortical thickness measures most highly correlated with the non-imaging variate were widely distributed in the parietal, temporal, frontal and visual cortex. Particularly, cortical thickness of the primary cortices showed a negative association with the behavioral variables while cortical thickness of associative cortices were positively associated (Figure 2, Supplementary Table 2). In the older group, higher age, blood pressure as well as better life satisfaction were the variables with the strongest negative associations to the sMRI dataset, while better cognitive functions remained among the most positive variables. In the sMRI dataset, lateral ventricles and orbitofrontal regions were identified as variables with the most negative associations to the behavioral variables, in contrast cortical thickness of the dorsomedial frontal regions, pre- and postcentral gyri showed the strongest positive associations.

### Sparse CCA between non-imaging and fMRI datasets

**Figure 3:**
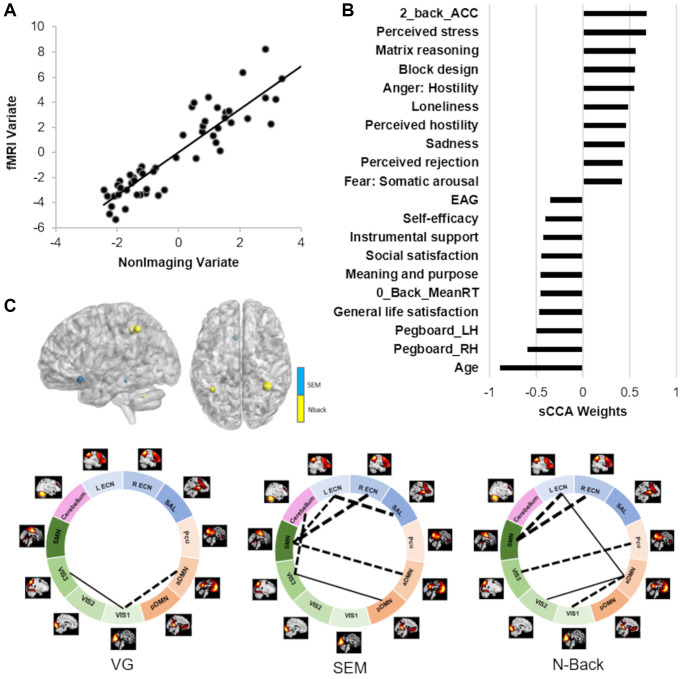
**Results of the sCCA between non-imaging and fMRI datasets across all participants.** (**A**) Significant correlation across all participants (r = 0.91, *p* = 0.0004). (**B**) Top behavioral–health variables most strongly associated with the imaging variate. (**C**) Top fMRI features most strongly associated with the non-imaging variate. Dashed lines between networks indicate negative contributions of the FNC; solid lines between networks indicate positive contribution of the FNC. Details in Supplementary Tables 3 and 5.

Across all participants, we found the sCCA significant between the non-imaging dataset and the fMRI variables (r = 0.91, *p* = 0.0004; Figure 3; Supplementary Table 3). In the non-imaging dataset, the highest positive weights were attributed to better cognitive ability (such as matrix reasoning and block design, higher accuracy during the fMRI tasks), and stronger report of emotions related to negative affect. In contrast, the highest negative weights were largely attributed to older age, lower motor dexterity (higher time to complete a pegboard with either right or left hand), psychological well-being, and higher estimated average glucose (EAG) rate (Figure 3, Supplementary Table 3). In the imaging dataset, the FNC measures mostly showed a negative association while the activation variables were positive contributors. Both the FNC and activation measures most highly correlated with the non-imaging dataset were collected during the n-back and Scene Encoding Memory (SEM) tasks. The networks most involved were largely related to executive function (ECN/SAL) and primary cortices (SMN/VIS).

**Figure 4:**
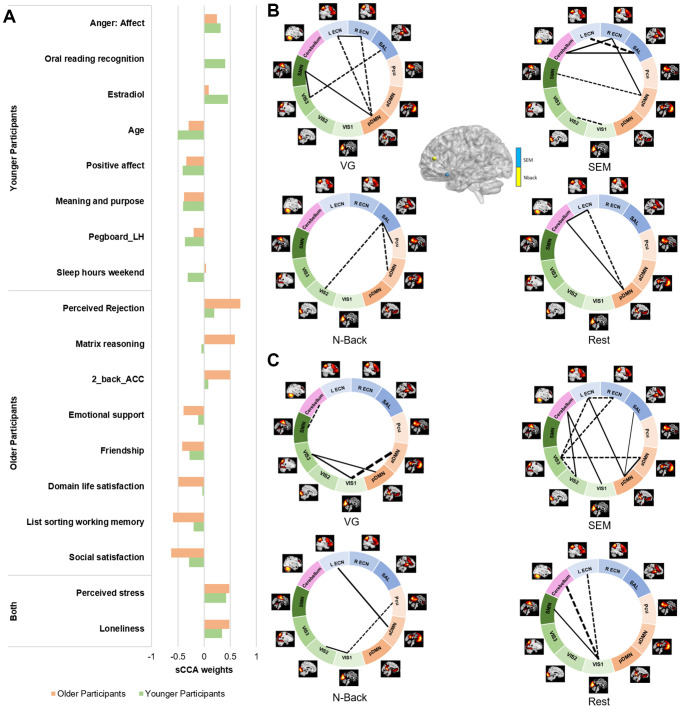
**Top 10 features contributing to the fMRI sCCA in younger and older groups, separately.** (**A**) Top behavioral–health variables most strongly associated with the imaging variate in each subgroup. (**B**) Top fMRI variables most strongly associated with the behavioral–health variate in the younger group. (**C**) Top fMRI variables most strongly associated with the behavioral–health variate in the older group. Dashed lines between networks indicate negative contributions of the FNC; solid lines between networks indicate positive contribution of the FNC. Details in Supplementary Tables 3 and 5.

We further identified the top contributors to the covariation between fMRI and behavior features within each age group (Figure 4, Supplementary Table 3). In the non-imaging dataset, the variables with the highest correlations with the fMRI dataset were different between the two groups. In the younger group, the top contributors were relatively similar to the results of the overall sCCA. The non-imaging variables with the strongest negative contributions were related to older age, motor dexterity, sleep quality during the weekend and positive affect; in contrast, estradiol rate, reading capacity and negative affect were positively associated with the fMRI dataset. In the imaging dataset, the FNC measures mostly showed a negative association while the activation variables were positive contributors. The FNC most highly correlated with the non-imaging dataset were collected during the Verb Generation (VG) and SEM tasks, largely involving networks related to executive function (ECN/SAL), primary cortices (SMN/VIS) and the default mode (aDMN, pDMN). In the older group, features related to better life were the variables with the strongest negative associations to the fMRI dataset, while better cognitive functions (higher accuracy and matrix reasoning) and negative affect remained among the most positive variables. In the fMRI dataset, no activation features showed strong associations with the behavioral dataset. In contrast, the FNC measures with the strongest associations were mostly identified during the SEM task, and involved the visual (VIS1/2/3), executive (ECNs) and default mode networks to a lower level.

### Reliability analyses

To assess whether our overall results were robust, we 1) performed leave-one out (LOO) analysis for every participant; 2) computed a redundancy-reliability score (Moser’s RR-score) for each overall sample sCCA [42]. The RR-score is a measure of the stability of the variable-to-variate correlations and indicates whether results can be expected to be reliable independent of sample composition (see detail in the methods). The LOO analyses indicated that both overall sCCAs were very stable and did not show outliers (the weights of each LOO analysis correlated above 0.95 with the sCCA weights in the full dataset). The RR-scores indicated that both overall sCCAs yielded reliable solutions (sMRI: median RR-score was 0.88, SD = 0.05; fMRI: median 0.87, SD = 0.11, Supplementary Figure 1). Lastly, when half the sample was randomly resampled 5000 times, both overall sCCAs yielded similar correlations. We then applied the weights of each sCCA resample to the remaining half of the sample (using it as a test-set). The mean (standard-deviation) of the scores indicated that the sCCA results held predictive value for both sCCAs (sMRI: r = 0.55 (0.11); fMRI: r = 0.67 (0.20)).

## DISCUSSION

The current study investigated the covariation between behavioral-health measures and brain structure and function across adulthood. While several previous studies have explored this relationship in adults, they typically focused on a limited age range, typically under 35 years (20~37 years for [7–9], 44–78 years for [17]). To our knowledge, the current study is among the first to investigate the multivariate associations among behavior and multimodal imaging phenotypes (structural MRI, task-based and resting-state fMRI) in a population that stretches across most of the adult lifespan (i.e., over 50 years; 20 to 73 years old). In line with our hypotheses, we found age-specific effects on brain-behavior covariations in healthy individuals. The impact of age was examined in two different and complementary methods: first, by examining the contribution of age in the brain-behavior associations across adulthood, and second by identifying the top variables that contributed to the covariations within older and younger age groups separately. Our results replicate previous findings focused on early adulthood, with the non-affective cognitive measures (e.g., IQ and other measures reflecting higher order cognitive functions) being the strongest positive contributions, and age and poorer motor dexterity being the most negative contributors to neuroimaging phenotypes [8, 9]. Beyond this general negative age-related impact, our findings revealed that such associations strongly vary by age group and neuroimaging phenotypes.

Across all participants, we found significant robust covariations between behavior and health measures and both brain structural morphometry and functional activation and connectivity measures. Better cognitive capacity typically had the strongest positive contribution to both brain structural and functional measures. In contrast, older age and slower motor dexterity showed a negative contribution. At the structural level, better cognition and younger age were related to thicker frontal and parietal cortices, among other widespread regions and larger subcortical volumes; while at the functional level, they were more strongly associated with greater activation and lower functional connectivity emerging from the brain networks supporting higher order cognitive functions, and particularly working memory (ECN, SAL). The degree of activation and functional connectivity of the working memory networks identified during the n-back task were among the strongest contributors to the non-imaging variables, which is consistent with both our previous findings [7, 9] and the fundamental role of these executive networks in everyday activity [43]. Interestingly, FNC and activations showed relatively opposite associations with behavior, where better cognition and younger age were associated with stronger activation and lower functional connectivity. In other words, older age was related to weaker activation in the memory networks and higher between-network functional connectivity. This finding supports the theory that healthy aging is associated with a progressive loss of functional integration and segregation of brain networks [20, 24, 44]. This opposition between activation and functional connectivity also points to the idea that these brain functional features are supported by different biological mechanisms [29].

In the fMRI sCCA, we found that the FNC features’ contributions to the covariation varied by cognitive states. In other words, the FNC during n-back, SEM, VG and rest did not contribute equally to the behavioral variables. Recent fMRI studies have demonstrated that the functional network organization of the brain is not stationary and rather reconfigures dynamically as a function of the cognitive state [45, 46], predicts general cognitive abilities [47], and varies by cognitive demands during the task [48]. Our results are in line with these findings and the recent study by Varangis et al. [48] which further described that age, task domain and performance on the in-scanner task impact between-network connectivity. Further, in the current study, the strongest FNC contributors to the behavioral variate were largely between brain networks supporting lower order function (i.e., networks related to sensory and motor processing such as SMN and VIS) versus those related to higher order functions (i.e., ECN, SAL and DMN). Variability in such extrinsic-intrinsic network interactions have been linked to higher order cognitive capacity [49–52], which is again supported by the current findings.

Surprisingly, we also found a relatively strong association of affective cognitive measures with both brain structural and functional features, where higher reported positive affect and life satisfaction was negatively associated with brain measures and negative affect and higher stress were rather positively associated. The exact meaning of such finding is unclear. While we used the t-scores for each emotional measure which are age- and sex-corrected, we cannot exclude the possibility that age ultimately influenced the measure. Affective cognition has been shown to change throughout the lifespan, with older adults reporting improvement in emotional experience and an increased frequency of positive feelings, relative to younger adults [53], which is also what we report (see Supplementary Table 4). Other neuroimaging studies have independently reported association between affective cognition and working memory network activation [7], functional network connectivity [8, 13, 54, 55] or structural morphometry [9]. It is also known from psychiatric studies that the activation of the working memory network is modulated by the level of depression and anxiety [56]. Overall, while the association between emotional well-being and brain functional and structural organization remains unclear and needs further investigation, we believe it likely reflects the complexity of the brain mechanisms behind emotional well-being and affective disorders across the lifespan [13, 57, 58].

When investigating each age group separately, we found that the strongest contribution of the behavioral variables to the brain features typically differed between the younger and older adults, which is in line with our hypotheses. Our findings support that such differences between early and late adulthood are likely related to the non-linear impact of age on brain structure [21, 22], function [24, 33] and cognition [39, 40]. First, in the current study, age was identified as a strong negative contributor to brain structural morphometry in the older but not in the younger adults, and vice-versa in the fMRI sCCA. This is consistent with the recent studies done by the ENIGMA-Lifespan working group which have demonstrated an almost null impact of age on structural brain morphometry during early and middle adulthood, before showing a strong negative effect in late adulthood [21, 22]. Second, in the sMRI sCCA, we found a relatively unexpected association in young adults where BMI and motor dexterity were among the top positive contributors to variation in brain structural morphometry. However, it is important to note that at the brain level, cortical thickness showed a heterogeneous pattern with both thinning of the primary cortices and thickening of associative cortices in association with these behavioral variables. Higher BMI has been previously associated with reduced functional integration of both visual and sensorimotor networks in young adults [59]. In this context, the current study suggests that such impact may be directly linked to underlying structural thinning of these primary regions that respond to perception of food images and tastes [60]. Third, in the fMRI sCCA, the major differences in contributions between the two age groups were identified at the neuroimaging level. While the major functional features associated with the behavioral dataset in the younger adults largely followed those from the overall analysis (i.e., large involvement of the executive networks to support higher cognitive capacity), the older adults showed a different pattern where behavior was rather supported by changes in functional connectivity emerging from the visual networks. These findings are in line with aging models [34, 40] that support the idea of an age-specific regional recruitment of primary cortices to support and maintain relatively preserved performance in older individuals. The STAC-r model supports the idea that preserved cognition in older adults is partially supported by good brain efficiency, which involves compensatory mechanisms through the mediation of primary networks, despite being less efficient than in their youthful state [32, 34]. With aging, this transition from executive to visual networks to support healthy behavior may reflect changes in biological mechanisms involving brain networks less impacted by aging [23, 34]. In fact, the overall negative impact of age on the functional integrity of the executive networks have been consistently reported [20, 23, 24, 44, 61], while the visual network is among the least impacted [23]. This relatively low impact of aging on the visual networks is likely related to their high structural-functional coherence [62], low inter- and intra-individual variability in functional activation [63], anatomical morphology [64, 65] and resting-state functional connectivity [66–68]. Together, the current findings indicate that, across adulthood: (a) there may be a progressive change in the functional support of healthy behavior and cognitive aging by recruiting more preserved networks such as the primary networks over executive control networks, as the latter become less efficient in late adulthood [44]; and (b) functional connectivity features may be more effective to highlight neural changes related to aging, than brain activation.

We found that variation in estradiol level was among the strongest variables associated with the fMRI dataset in the younger but not the older adults. We believe that this specific finding is related to a lack of variability in this hormone level in the older adults, as the majority of the older women reported being in menopause. However, this finding also underscores the impact of sexual hormones in brain functional organization in early adulthood.

Lastly, we expected a stronger contribution of health-related measures (such as physical activity, or EAG) to brain integrity in older adults but we did not reveal such effect. On the contrary, we found that BMI was a stronger contributor to brain structure in the younger group. With regard to physical activity, it is possible that only structured physical interventions have a significant impact on brain integrity and cognitive function [34], however, research studies on this topic remain relatively limited. While higher EAG contributed negatively to brain functional integrity across subjects, we did not see a specific impact within the older group. This lack of association in late adulthood may be related to the fact that the older adults had an EAG and HbA1C in the normal range. The overall negative association is consistent with literature showing that higher glucose ingestion is associated with negative changes in brain activity and connectivity [69], or that glucose fluctuations are linked to disrupted brain functional architecture and cognitive impairment [70]. This may also reflect that the impact of glycemia on brain function and cognition is independent of age.

While this study is among the first to investigate the covariation between different types of brain functional and structural features and a large series of behavioral and health variables across the adult lifespan, we must acknowledge specific limitations. First, our analyses were based on cross-sectional samples and not longitudinal data. As discussed by the STAC-r model, investigating the rate of within-subject cognitive change is essential to understand and identify brain integrity preservation versus compensatory mechanisms which may support preserved cognition in older adults [34]. To our knowledge, there is not yet any large longitudinal cohorts from older – or even younger- adults available to conduct sCCAs in a longitudinal fashion and identify features that predict future brain and cognitive preservation. Future studies should also investigate the impact of structured physical or cognitive interventions on brain-behavior covariations in late adulthood. Second, it will be important to test the reproducibility of our findings in a larger independent sample. It would also be interesting to investigate the impact of pathologic aging on the findings, although this might be challenging as clinical populations may not be able to complete all tasks in one session, as done in the current study. It is also likely that further differences between other subgroups could be revealed, especially in late adulthood (e.g., between participants aged 50-60 years-old and participants above 60, retired versus actively working). Such subgroups could also help identify and improve understanding of the origin of preserved cognition in late adulthood, including the role of neurobiology, compensatory processes, or a combination of both [34]. However, our sample size was too small to statistically test such differences. Lastly, we focused on specific tasks covering major cognitive functions and specific brain measures (FC and activation). Future studies should test other fMRI tasks and other neuroimaging measures (e.g., diffusion, graph theory) to determine their unique patterns of covariation with behavior, health and demographic characteristics.

The current study reinforces the importance of accounting for age in multivariate approaches when investigating the link between behavior and brain features, and confirms its non-linear impact across the lifespan. Our findings also highlight the complex interaction of 1) brain structure with age and variables related to stress and general well-being; and 2) brain activation with functional connectivity in their relationship with age, such that increased connectivity in older age may be used to compensate for the loss of brain activation in the networks supporting higher-order cognitive function. In summary, these data further underscore the need to use more multimodal approaches to investigate the impact of healthy aging on behavior, overall health and brain network organization.

## MATERIALS AND METHODS

### Participants

We recruited a total of 59 healthy individuals and divided them into two age groups: A younger adult group of 29 participants (mean age (SD) = 25.6 (3.4) years, age range: 20.2–32.8 years; 16 females) and an older adult group of 30 participants (mean age (SD): 62.5 (4.7) years, age range: 55.4–73.5 years; 19 females). The age cut-off for the older group (i.e., 55 years old minimum) was based on the age criterion used for the Alzheimer’s Disease Neuroimaging Initiative (ADNI) [71, 72]. Exclusion criteria included any chronic medical illness affecting central nervous system function, any neurological or psychiatric disorder, acute intercurrent illness, pregnancy, history of head trauma, current substance use disorder, and presence of any ferrous metal implant which may interfere with the MRI data acquisition. The study was approved by the Institutional Review Board for Research with Human Subjects at Boys Town National Research Hospital. Each participant provided written informed consent, and all participants completed the same protocol.

### Non-imaging data set

On the day of the scan, participants completed questionnaires and cognitive tests providing demographic information, personal and family medical history, IQ, cognitive scores, and physical activity. They also provided samples of saliva and blood after the MRI scan in order to extract their level of testosterone and estradiol, HbA1c and estimated average glucose (see detail in Supplementary Material). These variables were selected based on previous studies [7, 8]. Variables that were highly correlated (r > 0.85) or had low inter-subject variability (such as smoking habit, or family history of psychiatric disorders) were not included. A total of 59 variables were extracted and are detailed in Supplementary Table 5.

### MRI data acquisition

Participants were scanned on a 3T Siemens Prisma scanner using a 64-channel head coil. Anatomical and functional acquisitions were similar for all participants and adapted from the sequence parameters provided by the Human Connectome Project (HCP) [73]. Structural images were acquired using a T1-weighted, 3D magnetization-prepared rapid gradient-echo (MPRAGE) sequence with the following parameters: Repetition Time (TR) = 2400 ms, Echo Time (TE) = 2.22 ms, Field of View (FOV): 256 × 256 mm, matrix size: 320 × 320, 0.8 mm isotropic resolution, Inversion Time (TI) = 1000 ms, 8 degree-flip angle, bandwidth = 220 Hz/Pixel, echo spacing = 7.5 ms, in-plane acceleration GRAPPA (GeneRalized Autocalibrating Partial Parallel Acquisition) factor 2, total acquisition time ~7 min. Participants also completed three tasks and one resting-state fMRI scans, using an identical multi-band T2^*^ sequence with the following acquisition parameters: TR = 800 ms, TE = 37 ms, voxel size = 2 × 2 × 2 mm^3^, echo spacing 0.58 ms, bandwidth = 2290 Hz/Pixel, number of axial slices = 72, multi-band acceleration factor = 8. The numbers of volumes collected were: 450 for the n-back task, 375 for the verb generation task, 340 for the scene encoding memory task, and 460 for the resting-state fMRI.

### Task fMRI scans: description of the tasks

Each participant completed a n-back, a SEM and a VG task. These tasks were specifically chosen because: (1) they are associated with cognitive functions among the most commonly investigated in fMRI, and (2) they have been demonstrated as reliable and robust activators of specific brain networks. In short, the n-back fMRI task that we used was developed for the HCP and aimed to activate the network supporting working memory ability [74]. It has a block design incorporating alternating experimental (2-back) and sensorimotor control (0-back) conditions. The SEM task was based on the fMRI task developed by Binder et al. known to be a robust activator of bilateral mesial temporal lobe (MTL) structures [75]. This task has a block design incorporating alternating experimental (scene encoding) and control conditions. During the scene encoding condition, the participant was required to identify a given scene as indoor or outdoor from the variety of landscapes or home/office photos presented. This binary judgment ensured attentional engagement and full encoding of the scene. During the control condition, the participant was presented with scrambled pictures divided into two halves and was required to identify if the two halves were identical. Lastly, the VG task aims to activate a network supporting language function. The task has a block design incorporating alternating experimental (verb generation) and control (passive viewing of ‘xxxx’) conditions. In the active condition, participants were instructed to covertly generate an action word in response to a viewed object noun presented on a screen. This task has been extensively used in clinical fMRI protocols for epilepsy to localize and lateralize the language network [76, 77]. A detailed description of each task can be further found in Supplementary Material.

In addition, a resting-state fMRI scan was collected during which participants were instructed to remain still and keep their eyes on a fixation cross throughout the scan. For all participants, the resting-state scan was collected before the three tasks in order to avoid cognitive interference from the tasks on the resting-state mental activity.

### Structural preprocessing

In each individual sMRI dataset, we used FreeSurfer image analysis suite (v.6.0) (http://surfer.nmr.mgh.harvard.edu/) to derive 68 cortical thickness measures and 18 subcortical volumetric measures from the Desikan atlas [78]. The outputs were quality controlled using protocols developed by the ENIGMA consortium (http://enigma.ini.usc.edu/). Prior to being entered in further analyses, subcortical volumes were adjusted for variation in intracranial volume.

### FMRI preprocessing

The resting-state and task-based fMRI data were preprocessed using SPM12 and the DPABI Toolbox [79]. For all runs, preprocessing procedures included motion correction to the first volume with rigid-body alignment; co-registration between the functional scans and the anatomical T1-weighted scan; spatial normalization of the functional images into Montreal Neurological Institute (MNI) stereotaxic standard space; and spatial smoothing within the functional mask with a 6-mm at full-width at half-maximum Gaussian kernel. For the functional connectivity analyses only (including the resting-state fMRI data and the 3 task-based fMRI data sets), additional steps were applied for each dataset including linear detrending, regression of motion parameters, their derivatives (24-parameter model, [80]), and the scrubbing parameters [81], as well as white matter (WM), and cerebrospinal fluid (CSF) time series (using a component based noise reduction method, 5 principal components [82]).

### Subject exclusion and quality control

A total of six participants were excluded from group analyses because of right hemispheric dominance for language function based on the VG task (*n* = 2), incomplete collection of the fMRI tasks (i.e., one missing task, *n* = 2), mini-mental state (MMSE) inferior to 25 (*n* = 1), and excessive head motion during the resting-state fMRI sequence (*n* = 1). The final sample was 27 younger participants (mean age (std) = 25.5 (3.4) years, 14 females) and 26 older participants (mean age (std) = 62.1 (4.5) years, 16 females). Demographic and cognitive details on each group are provided in Supplementary Table 4.

### FMRI task activation

For each task, general linear model analyses were implemented using Statistical Parametric Mapping (SPM12). For each task, the preprocessed single-participant images were analyzed in a similar fashion, using a linear convolution model, with vectors of onset representing the experimental (n-back: 2-back; VG: verb generation; SEM: indoor/outdoor categorization) and the control condition (n-back: 0-back; VG: passive viewing of “xxxxx”; SEM: scramble pictures). In each participant, images were produced for the active versus control contrast. In each model, the six movement parameters were entered as nuisance covariates. Serial correlations were removed using an AR(1) model. A high-pass filter (128 seconds) was applied to remove low-frequency noise. At the group level, brain regions activated were identified using a random-effects one-sample *t*-test of the single-participant contrast images for each task. Age and sex were entered as covariates. The statistical threshold was set up at a height threshold of a minimum of T > 3.3 at the whole brain level, which refers to a maximum *p* < 0.001 at the voxel level, and cluster size >50 voxels. Furthermore, at the cluster level, activation peaks were only selected if they had a T-value >4.5. The threshold was adapted for each task as they differ in statistical power (detail in Supplementary Material, Supplementary Tables 6–8). Using the above parameters, for the n-back task, we identified: 12 regions localized in the dorsolateral prefrontal cortex (dlPFC), inferior parietal lobule, supplementary motor area, precuneus, thalamus and cerebellum (Supplementary Table 6 and Supplementary Figure 2). For the VG task, we identified 8 regions localized in the left inferior frontal gyrus, supplementary motor area, inferior parietal lobe, left hippocampus, and cerebellum (Supplementary Table 8, Supplementary Figure 3A). For the SEM task, we identified 9 regions localized in the fusiform and lingual gyri, retrosplenial cortex, orbitofrontal cortex, and lateral occipital cortex (Supplementary Table 7, Supplementary Figure 3B). For each task, the regions identified were consistent with those expected based on previous studies [7, 76, 83–85].

### Extraction of the activation peaks

Using the Marsbar toolbox, we created 4-mm radius spherical volumes-of-interest (VOIs) centered on each group peak coordinates of each network node and extracted the mean beta values for each respective contrast in each individual. In total, 29 activation variables per individual were entered in the sCCAs. Effect sizes for each peak activation measure between younger and older groups are reported in Supplementary Figure 4.

### Independent component analysis

**Figure 5:**
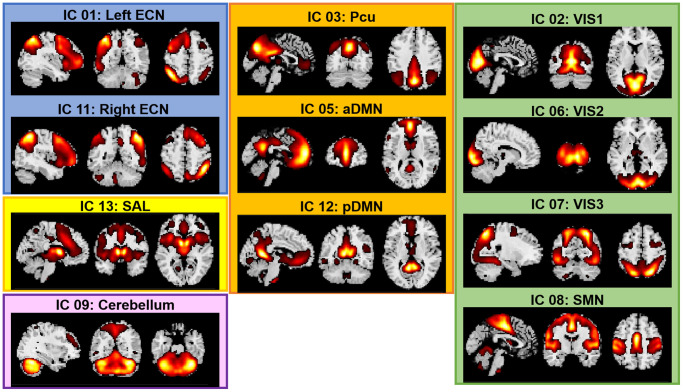
**Spatial maps of the 11 networks identified across fMRI sessions and participants.** Abbreviations: ECN: Executive Control Network; DMN: Default Mode Network; SAL: Salience Network; SMN: Sensorimotor Network; VIS: Visual Network; a: anterior; p: posterior; Pcu: Precuneus.

We conducted a group-based Independent Component Analysis (ICA) to simultaneously identify the major brain networks across the four functional runs because this approach: (a) is fully data-driven (i.e., does not require *a priori* seeds), (b) has been widely demonstrated as among the most robust approaches to artifacts with minimal assumptions, and (c) allows the identification of cortical, subcortical and cerebellar networks [86]. Group ICA of fMRI Toolbox (GIFT, https://trendscenter.org/software/, version 3.0c) was used to extract spatially independent components (ICs) across all the fMRI datasets and all the participants [87, 88]. The fMRI data from the three tasks and the resting-state from all participants were concatenated into a single dataset and reduced using two stages of principal component analysis (PCA) [87]. We extracted 20 ICs by using Infomax algorithm [89]. The Infomax algorithm generated a spatial map and a time course of the source signal changes for each IC. This analysis was repeated 20 times using ICASSO for assessing the repeatability of ICs [90] and the 20 most reliable components were identified as the final group-level components. IC’s time courses and spatial maps were back-reconstructed for each participant using Group information guided (GIG)-ICA [91] implemented in the GIFT Toolbox. GIG-ICA was chosen because it has been shown as more sensitive to group differences [92]. Finally, the 20 ICs were evaluated to identify functionally relevant brain networks. The criteria for identifying the networks were: (1) the peak clusters of a network should be in the grey matter, and (2) there should be minimal overlap with known vascular, susceptibility, ventricular and edge regions. Following this selection procedure, 11 networks were selected for functional connectivity analyses (Figure 5).

### Functional connectivity analyses

Upon completing the group ICA and the selection of the 11 networks, we extracted FNC correlations for each pair of networks and within each dataset separately (each of the three tasks and the resting-state), using the GIFT Toolbox. Before FNC computation, subject-specific time courses were detrended and despiked using 3dDespike (AFNI, 1995), then filtered using a fifth-order Butterworth low-pass filter with a high frequency cutoff of 0.15 Hz. This resulted in 55 FNC measures per dataset and per subject. Effect sizes for each FNC measure between younger and older groups are reported in Supplementary Figure 4.

### Sparse canonical correlation analyses

We conducted sCCAs to determine the covariation patterns between behavioral features with sMRI and with fMRI neuroimaging measures.

We used an sCCA approach with an L1-norm penalty [35], using a MATLAB script available online [93]. To do this, the sCCA specifies linear combinations (pairs of canonical variates) of variables in the behavioral-health dataset and variables in the neuroimaging dataset that best express the maximal correlation (i.e., canonical correlation) between the two datasets. The correlations between the canonical variates are the canonical correlations. To achieve this, the algorithm groups variables from either side into component pairs/dimensions, which are referred to as modes in the present paper. Instead of a classic (non-sparse) canonical correlation analysis, we conducted sCCA because this analysis (a) permits the inclusion of more features than observations and (b) allows stronger inferences regarding the contribution of individual variables (for similar approaches, see [7, 9, 55]; also see reviews by Zhuang et al. [36], Wang, Smallwood [94]).

Prior to being entered into the sCCAs, both imaging and behavioral-health variables were z-standardized. The neuroimaging measures were combined into two datasets: the cortical thickness and subcortical volumes on the one hand (total *n* = 86, referred to as the “sMRI dataset”), and the activation-related betas from each task and FNC variables during each fMRI run on the other hand (total *n* = 249, referred to as the “fMRI dataset”). The non-imaging measures included 59 variables listed in Supplementary Table 5. For each analysis (i.e., non-imaging versus sMRI dataset, and non-imaging versus fMRI dataset), we selected the optimal sparse criteria combination based on the parameters that corresponded to the values of the model that maximized the sCCA correlation value. We then computed the optimal sCCA model and determined its significance using permutations. Accordingly, the non-imaging dataset was permuted 5,000 times before undergoing the exact same analysis as the original data. The *P*-value was defined as the number of permutations that resulted in a higher correlation than the original data divided by the total number of permutations. Thus, the *P*-value is explicitly corrected for multiple testing as it is compared against the null distribution of maximal correlation values across all estimated sCCAs. The threshold for statistical significance for each analysis was set at *P* < 0.05 after 5,000 permutations. When the sCCA was significant, we investigated the contribution weight of each variable in the whole group, and in each subgroup separately (on both the imaging and behavioral data sets). To do so, we computed Pearson’s correlations between each variable and the mode of the opposing pattern (that is, each non-imaging variable to mode of the neuroimaging dataset and vice versa).

### Subsidiary analyses

To assess whether our overall results were robust, we 1) performed leave-one out (LOO) analysis for every participant; 2) computed a redundancy-reliability score (Moser’s RR-score) for each overall sample sCCA [42]. The RR-score is a measure of the stability of the variable-to-variate correlations and indicates whether results can be expected to be reliable independent of sample composition. The RR-score is based on a training-test set approach and essentially measures whether test sets have similar associations between variables and variates, whereby results with high RR-scores can be assumed to be truly carried by the entire sample and not to be dependent on a specific subset of the population that may not be reliably reproduced if one were to replicate the study. It ranges between 0 and 1; a value of 0 indicates no correlation between the canonical correlations generated from the randomly resampled 5000 test-sets created during the reliability analyses and a value of 1 indicates complete agreement. In the present study, 5,000 splits of training and test sets were performed in order to calculate the mean RR-score. The RR-score is thus an index of similarity between all test-sets. RR-scores that are close to 1 indicate that a test-set would have yielded very similar results as the mean of test-sets. We report the median RR-score and the standard-deviation for each overall sCCA. And 3) We randomly split the sample in half 5,000 times (creating a training and a test sets), performed an sCCA on each of these training sets and then applied the identified weights to the other half of the sample (the test set). This allowed us to gain information on whether sCCA derived correlations in this study were likely to be dependent on this specific sample or not.

The code to compute the Moser’s RR score is available at: https://github.com/domamo/Matlab-code-and-example-to-calculate-RR-score-as-related-to-Moser-et-al-2018.

## Supplementary Materials

Supplementary Materials and Methods

Supplementary Figures

Supplementary Tables
