# Supplementary material for: Multivariate patterns of brain-behavior associations across the adult lifespan
Source: Aging (Albany NY). 2022 Jan 10;14(1):161–94. doi: 10.18632/aging.203815 (PMC8791210; doi:10.18632/aging.203815)
Supplement: Supplementary Materials and Methods [file aging-14-203815-s001.pdf]

# SUPPLEMENTARY MATERIALS AND METHODS

## Non-imaging dataset collection

Participants completed a series of questionnaires regarding demographic information, medical history for both self and biological family members, and lifetime of experiences. In order to measure their cognitive capacity, they completed the Mini-Mental Status Examination (MMSE), WASI-II [1], and the NIH Toolbox for Adult, including cognition, emotion and motor batteries (https://www.healthmeasures.net/explore-measurement-systems/nih-toolbox/intro-to-nih-toolbox). Lastly, immediately after the fMRI scan, samples of saliva and blood were collected. From the blood sample, HbA1c and estimated average glucose (EAG) levels were extracted. The saliva sample was analyzed by the University of Nebraska-Lincoln Salivary Bioscience Laboratory to measure the level of estradiol and testosterone. A total of 59 non-imaging variables were extracted and described in Supplementary Table 5.

## Task description

### *N-back task*

The task was the one developed by the Human Connectome Project (HCP), and has a block design incorporating alternating experimental (2-back) and sensorimotor control (0-back) conditions [2]. At the start of a block, a written cue (lasting 2.5 seconds) informed participants about the type of condition to follow (2-back or 0-back) and the designated target stimulus for the sensorimotor control condition. Four different stimulus types (faces, places, tools and body parts) were presented in separate blocks. Each stimulus was presented for 3 seconds, followed by a 500 ms inter-stimulus interval. In the 2-back trials participants were asked to respond by pressing a button whenever the current stimulus was the same as the one presented 2 trials back. In the 0-back condition, participants were asked to respond by pressing a button each time they saw the designated stimulus. The run contained 8 blocks of 10 trials, each lasting 35 seconds, and 4 fixation blocks each lasting 15 seconds. Performance features (accuracy and reaction time) were monitored and collected during fMRI scanning.

### *Verb Generation (VG) task*

This task is known to be a robust activator of the language network [3, 4]. Participants were instructed to covertly generate an action word in response to a viewed concrete noun presented on a screen. Each word was presented for 3 s, within a 30-second block. These blocks were alternated with passive viewing of a central stimulus (#####) in epochs of 30 s for a total of 5 min.

### *Scene Encoding Memory (SEM) task*

This task was adapted from the task created by Binder et al. [5] known to be a robust activator of bilateral mesial temporal lobe (MTL) structures. A block-design format was utilized. During the scene encoding condition, the participant was required to identify a given scene as indoor or outdoor from the variety of landscapes or home/office photos presented. This binary judgment ensured attentional engagement and full encoding of the scene. During the control condition, the participant was presented with scrambled pictures divided into two halves, and required to identify if the two halves were identical. This control condition allowed us to subtract the visuo-perceptual and decision-making aspects of task performance. The session contained four blocks of scene encoding and four blocks of the control condition, each block for 28 s in duration. Each session started and ended with a 28-second period of blank screen. For each of the scene encoding and control blocks, 8 stimuli (e.g., scene/scrambled picture) were presented, each for 3 s, interleaved with a 0.5 s of blank screen. The participant discriminated indoor scenes from outdoor scenes (indoor session), and vice versa in the other session (outdoor session), with the order of sessions randomly counterbalanced across participants. 60% of the stimuli presented during the scene encoding blocks within that session were the targets, and the rest (40%) were nontargets; while, 60% of the stimuli presented during the control blocks contained scrambled pictures (halves) that were identically matched (to be referred to as targets as well), and the other 40% were not (i.e., nontargets). All visual stimuli were presented only once during the entire study. The participants were instructed to press a button with their index finger for target pictures and another button with their middle finger for non-target pictures. Performance features (accuracy and reaction time) were monitored and collected during fMRI scanning.

## Task activation

### *N-back task*

See Supplementary Table 6 and Supplementary Figure 2.

### *Verb Generation*

See Supplementary Table 8 and Supplementary Figure 3.

### *Scene Encoding Memory*

See Supplementary Table 7 and Supplementary Figure 3.

### Difference in brain activity between younger and older participants

We computed the effect size for each functional measure (Cohen's D) between younger and older participants (Supplementary Figure 4). For both VG and N-back tasks, older participants showed lower activation, while for the SEM task, older showed higher activation, than the younger participants. For the FNC measures, older participants showed a large majority of lower FC, compared to the younger participants.

### Sparse canonical correlation analyses (sCCAs)

*Non-imaging dataset vs. sMRI dataset*
See Supplementary Tables 1 and 2.

*Non-imaging dataset vs. fMRI dataset*
See Supplementary Table 3.

### Reliability analyses

See Supplementary Figure 1.

## REFERENCES


1. Wechsler D. Wechsler Abbreviated Scale of Intelligence | Second Edition. San Antonio, TX: NCS Pearson. 2011.

2. Barch DM, Burgess GC, Harms MP, Petersen SE, Schlaggar BL, Corbetta M, Glasser MF, Curtiss S, Dixit S, Feldt C, Nolan D, Bryant E, Hartley T, et al, and WU-Minn HCP Consortium. Function in the human connectome: task-fMRI and individual differences in behavior. Neuroimage. 2013; 80:169–89. https://doi.org/10.1016/j.neuroimage.2013.05.033 PMID:23684877

3. Doucet GE, He X, Sperling MR, Sharan A, Tracy JI. From "rest" to language task: Task activation selects and prunes from broader resting-state network. Hum Brain Mapp. 2017; 38:2540–52. https://doi.org/10.1002/hbm.23539 PMID:28195438

4. Binder JR, Frost JA, Hammeke TA, Bellgowan PS, Springer JA, Kaufman JN, Possing ET. Human temporal lobe activation by speech and nonspeech sounds. Cereb Cortex. 2000; 10:512–28. https://doi.org/10.1093/cercor/10.5.512 PMID:10847601

5. Binder JR, Bellgowan PS, Hammeke TA, Possing ET, Frost JA. A comparison of two FMRI protocols for eliciting hippocampal activation. Epilepsia. 2005; 46:1061–70. https://doi.org/10.1111/j.1528-1167.2005.62004.x PMID:16026558
