# Supplementary material for: Multivariate patterns of brain-behavior associations across the adult lifespan
Source: Aging (Albany NY). 2022 Jan 10;14(1):161–94. doi: 10.18632/aging.203815 (PMC8791210; doi:10.18632/aging.203815)
Supplement: Supplementary Figures [file aging-14-203815-s002.pdf]

SUPPLEMENTARY FIGURES

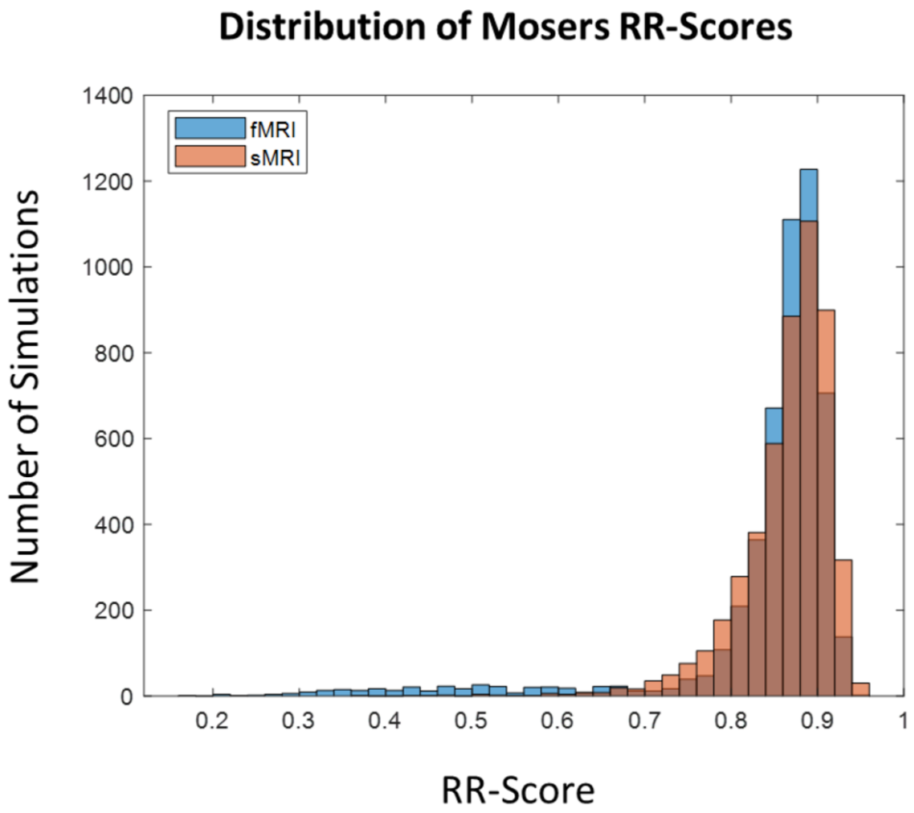

Supplementary Figure 1. Distribution of the redundancy-reliability (RR) score for each sCCA, over the 5,000 simulations.

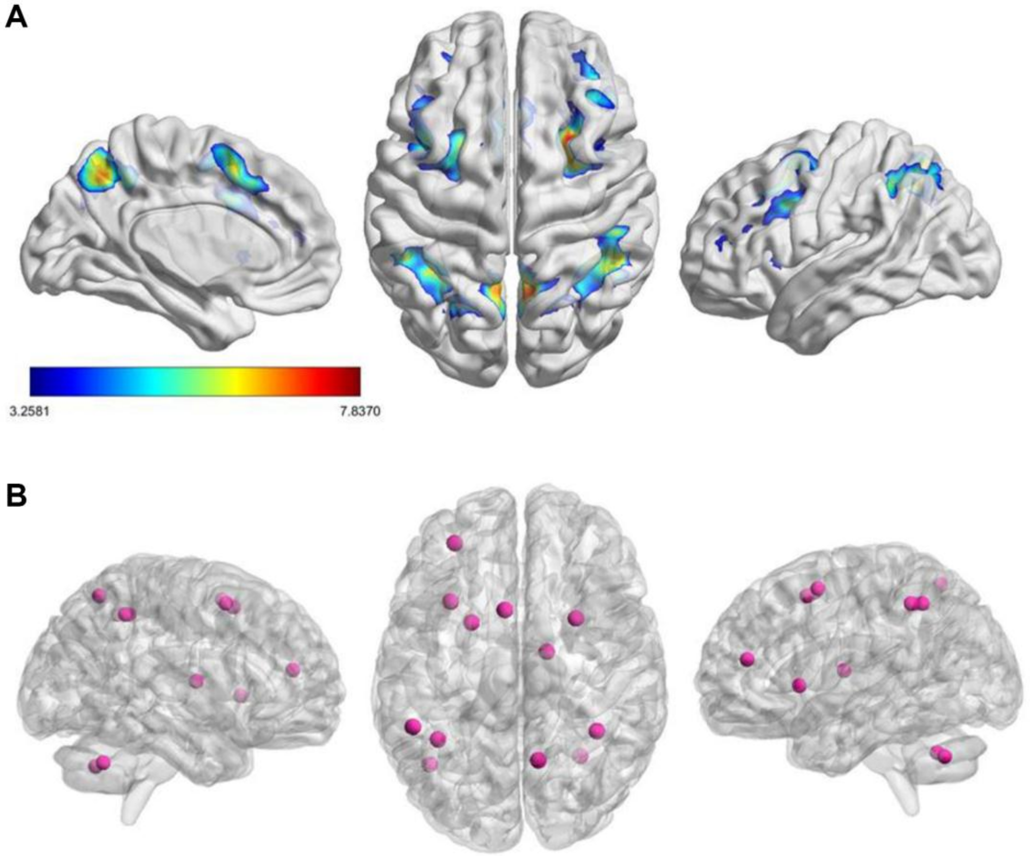

Supplementary Figure 2. Working memory network identified in the 2back-0back contrast across all participants, during the n-back fMRI task. (**A**) Activation map, (**B**) 12 nodes reflecting the activation peaks (coordinates are provided in Supplementary Table 6).

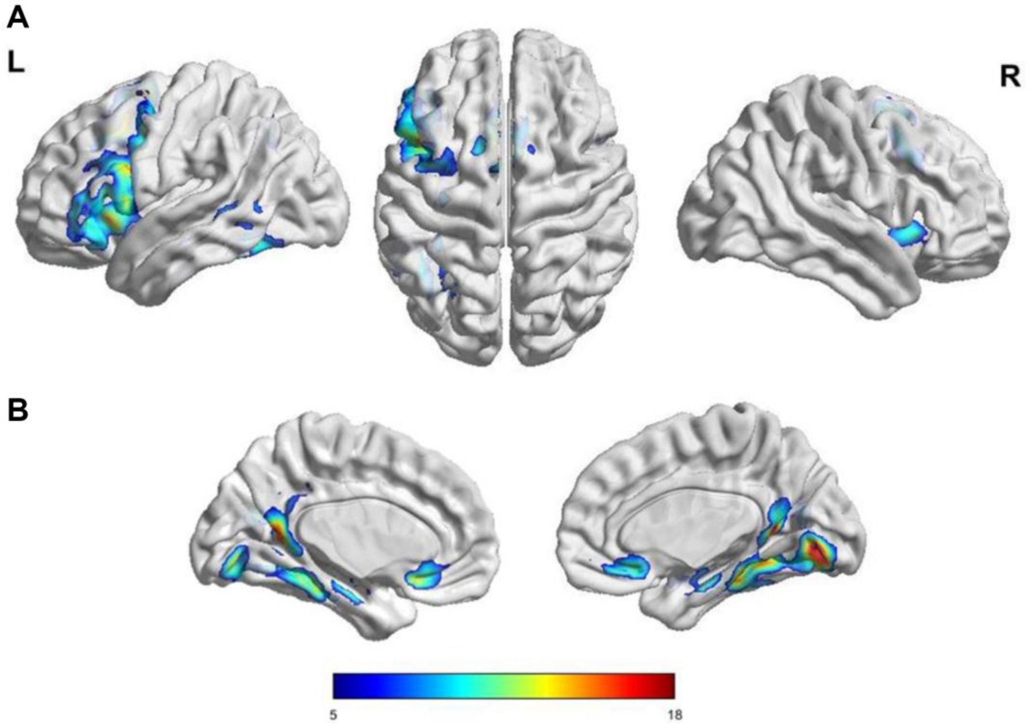

Supplementary Figure 3. Activation Maps for the Verb Generation (**A**) and Scene Encoding Memory Task (**B**).

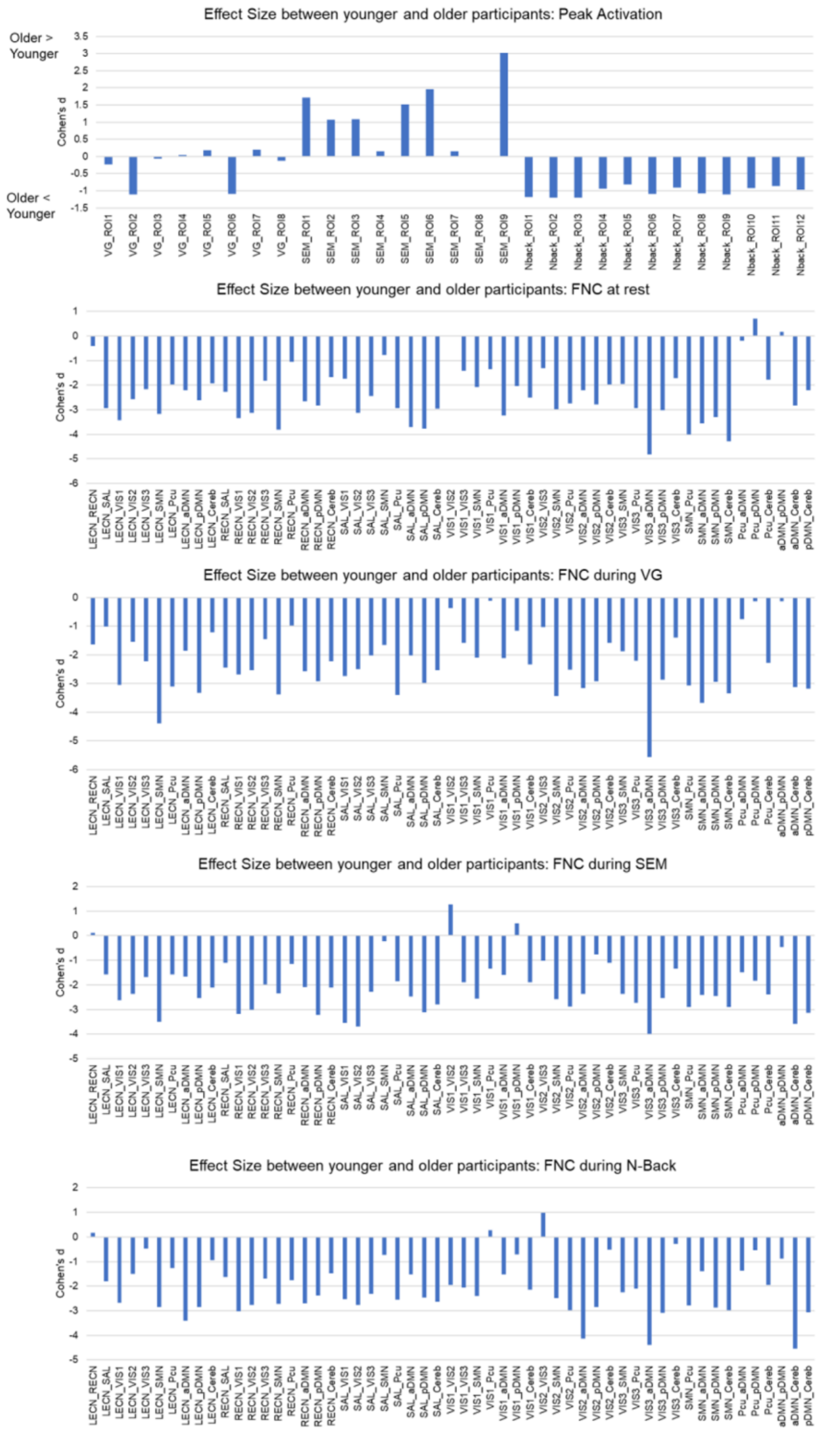

Supplementary Figure 4. Effect size between younger and older participants for each functional variable. A positive score reflects higher activation/connectivity in the older than in the younger participants.
