# Supplementary material for: Multivariate patterns of brain-behavior associations across the adult lifespan
Source: Aging (Albany NY). 2022 Jan 10;14(1):161–94. doi: 10.18632/aging.203815 (PMC8791210; doi:10.18632/aging.203815)
Supplement: Supplementary Tables [file aging-14-203815-s003.pdf]

SUPPLEMENTARY TABLES

Supplementary Table 1. Canonical weights of the non-imaging variables to the sMRI variate.

Non-imaging Variable	All participants: sCCA Weights	Younger participants only: sCCA Weights	Older participants only: sCCA Weights
**Semantic fluency** (animal_name)	**r = 0.53**	r = 0.34	**r = 0.44**
**Perceived stress** (emo_psff_t)	**r = 0.51**	r = 0.11	r = 0.22
**Anger-Hostility** (emo_ahff_t)	**r = 0.44**	r = 0.003	**r = 0.32**
**Sadness** (emo_sff_t)	**r = 0.43**	r = 0.23	r = 0.23
**Block_Design**	**r = 0.43**	r = −0.29	**r = 0.39**
**2_back_ACC**	**r = 0.42**	r = −0.12	r = 0.03
**Apathy** (emo_aff_t)	**r = 0.36**	**r = 0.43**	r = 0.21
**Loneliness** (emo_lff_t)	**r = 0.35**	r = 0.08	r = 0.22
**Perceived hostility** (emo_phff_t)	**r = 0.34**	r = 0.05	r = 0.13
**0_back_ACC**	**r = 0.34**	r = −0.08	r = 0.07
**Matrix_Reasoning**	r = 0.33	r = −0.11	r = 0.1
**Apathy** (emo_aff_t)	r = 0.32	r = 0.06	r = 0.18
**Fear-Affect** (emo_faff_t)	r = 0.31	r = −0.06	**r = 0.28**
**SEM_Scramble_ACC**	r = 0.3	r = −0.28	r = 0.2
**Crystalized intelligence** (cog_cc_t)	r = 0.25	**r = 0.37**	r = −0.19
**Physical activity**	r = 0.21	r = 0.13	r = −0.27
**Testosterone**	r = 0.21	r = −0.01	r = 0.03
**Perceived rejection** (emo_prff_t)	r = 0.2	r = 0.36	r = −0.18
**Oral reading recognition** (cog_orr_t)	r = 0.2	r = 0.03	r = −0.04
**Verbal fluency** (total_FAS)	r = 0.2	r = −0.1	r = 0.22
**Weekend night sleep**	r = 0.18	r = 0.07	r = 0.15
**Sex**	r = 0.17	r = 0.34	r = −0.02
**MMSE_Total**	r = 0.16	r = −0.14	r = 0.21
**Fear-Somatic arousal** (emo_fsaff_t)	r = 0.15	r = −0.19	r = −0.21
**Similarities**	r = 0.09	r = −0.28	r = 0.2
**Vocabulary**	r = 0.09	r = −0.17	r = 0.17
**SEM_Scramble_RT**	r = 0.08	r = 0.32	**r = 0.27**
**Anger-Physical aggression** (emo_apaff_t)	r = 0.06	r = −0.12	r = −0.23
**FSIQ**	r = 0.06	r = −0.31	r = 0.21
**Picture sorting memory** (cog_psm_t)	r = 0.06	r = −0.22	r = −0.15
**Estradiol**	r = 0.04	r = −0.01	r = −0.2
**Weeknight sleep**	r = 0.04	r = 0.01	r = 0.05
**Number of alcoholic drinks**	r = 0.01	r = −0.34	r = 0.06
**Picture vocabulary** (cog_pv_t)	r = 0.002	r = 0.21	r = −0.23
**BMI**	r = −0.01	**r = 0.47**	r = 0.15
**Dimensional card change sort** (cog_dccs_t)	r = −0.06	r = −0.26	r = −0.04
**SEM_Scene_RT**	r = −0.07	**r = 0.38**	r = 0.16
**Pattern comparison** (cog_pcps_t)	r = −0.08	r = −0.38	r = −0.06
**SEM_Scene_ACC**	r = −0.09	r = −0.36	r = 0.12
**Fluid intelligence** (cog_fc_t)	r = −0.12	**r = −0.49**	r = −0.21
**Flanker inhibitory control and attention** (cog_fica_t)	r = −0.15	r = −0.26	r = −0.25
**EAG**	r = −0.16	r = 0.25	r = 0.05

**HBA1C**	r = −0.16	r = 0.25	r = 0.04
**Friendship** (emo_fff_t)	r = −0.21	**r = −0.5**	**r = −0.37**
**List sorting working memory** (cog_lswm_t)	r = −0.26	**r = −0.51**	r = −0.12
**Positive affect** (emo_paff_t)	r = −0.27	r = −0.04	r = −0.32
**High blood pressure**	r = −0.31	r = −0.11	**r = −0.37**
**0_back_RT**	r = −0.32	r = 0.22	r = 0.08
**Pegboard_RH**	r = −0.33	**r = 0.39**	r = −0.02
**2_back_RT**	**r = −0.34**	r = 0.19	r = −0.17
**Instrumental support** (emo_isff_t)	**r = −0.36**	r = −0.38	r = −0.03
**Pegboard_LH**	**r = −0.37**	r = −0.03	r = −0.09
**Social satisfaction** (emo_sss_t)	**r = −0.4**	r = −0.45	r = −0.2
**Self-efficacy** (emo_secat_t)	**r = −0.42**	r = −0.31	r = −0.23
**Domain life satisfaction** (emo_dslsff_raw)	**r = −0.42**	**r = −0.48**	r = −0.3
**Emotional support** (emo_esff_t)	**r = −0.42**	**r = −0.51**	r = −0.22
**Meaning and Purpose** (emo_mpff_t)	**r = −0.42**	r = −0.19	**r = −0.37**
**General life satisfaction** (emo_glsff_t)	**r = −0.55**	r = −0.26	**r = −0.51**
**Age**	**r = −0.74**	r = 0.15	**r = −0.57**

Variables are described in Supplementary Table 5. Top contributions shown in the main text are highlighted in bold.

Supplementary Table 2. Canonical weights of the sMRI variables to the non-imaging variate.

**Region**	**All Subjects: sCCA Weights**	**Younger Participants Only: sCCA Weights**	**Older Participants Only: sCCA Weights**
Right Superior Frontal Thickness	**r = 0.74**	**r = 0.48**	**r = 0.51**
Left Superior Frontal Thickness	**r = 0.72**	**r = 0.39**	**r = 0.48**
Left Pars Opercularis Thickness	**r = 0.7**	r = 0.21	**r = 0.51**
Right Pars Opercularis Thickness	**r = 0.64**	r = 0.17	r = 0.4
Right Insula Thickness	**r = 0.63**	r = 0.07	r = 0.32
Right Superior Temporal Thickness	**r = 0.63**	r = −0.03	**r = 0.48**
Left Precentral Thickness	**r = 0.62**	r = −0.05	**r = 0.46**
Left Pars Triangularis Thickness	**r = 0.6**	r = 0.19	r = 0.41
Left Superior Temporal Thickness	**r = 0.58**	r = 0.19	r = 0.31
Right Paracentral Thickness	**r = 0.58**	r = 0.01	**r = 0.51**
Right Pars Triangularis Thickness	**r = 0.58**	r = 0.01	r = 0.32
Left Bankssts Thickness	**r = 0.55**	**r = 0.28**	r = 0.29
Left Caudal Middle Frontal Thickness	**r = 0.55**	**r = 0.37**	r = 0.24
Right Precuneus Thickness	**r = 0.54**	**r = 0.31**	r = 0.38
Right Precentral Thickness	**r = 0.52**	**r = −0.25**	**r = 0.45**
Right Caudal Middle Frontal Thickness	**r = 0.52**	r = 0.18	r = 0.26
Right Superior Parietal Thickness	**r = 0.52**	r = 0.01	**r = 0.53**
Left Inferior Parietal Thickness	r = 0.5	r = 0.35	r = 0.34
Right Supramarginal Thickness	r = 0.49	r = 0.07	r = 0.28
Left Rostral Anterior Cingulate Thickness	r = 0.49	r = 0.22	r = 0.13
Left Insula Thickness	r = 0.49	r = 0.09	r = 0.06
Left Postcentral Thickness	r = 0.47	r = −0.07	**r = 0.62**
Right Inferior Parietal Thickness	r = 0.47	r = 0.2	r = 0.25
Left Thalamus Volume	r = 0.47	r = −0.1	r = 0.1

Left Supramarginal Thickness	r = 0.46	r = 0.1	r = 0.16
Left Accumbens Volume	r = 0.45	r = 0.02	r = 0.22
Right Putamen Volume	r = 0.44	r = 0.09	r = 0.05
Left Middle Temporal Thickness	r = 0.44	r = −0.03	r = 0.17
Left Pallidum Volume	r = 0.43	**r = −0.19**	r = 0.39
Left Superior Parietal Thickness	r = 0.42	r = 0.28	**r = 0.47**
Left Amygdala Volume	r = 0.42	r = −0.03	r = 0.34
Right Middle Temporal Thickness	r = 0.42	**r = −0.21**	r = 0.01
Left Precuneus Thickness	r = 0.41	r = 0.19	r = 0.4
Right Rostral Middle Frontal Thickness	r = 0.41	**r = 0.33**	r = 0.17
Left Putamen Volume	r = 0.39	r = 0.01	r = 0.21
Right Pallidum Volume	r = 0.38	r = −0.1	**r = 0.45**
Left Rostral Middle Frontal Thickness	r = 0.38	r = 0.13	r = 0.29
Left Posterior Cingulate Thickness	r = 0.36	r = −0.06	r = −0.04
Right Thalamus Volume	r = 0.33	r = −0.1	r = 0.12
Right Postcentral Thickness	r = 0.33	r = −0.17	**r = 0.44**
Right Posterior Cingulate Thickness	r = 0.33	r = 0.02	r = 0.04
Right Lateral Orbitofrontal Thickness	r = 0.32	r = 0.27	r = −0.06
Right Rostral Anterior Cingulate Thickness	r = 0.32	r = 0.21	r = 0.08
Right Accumbens Volume	r = 0.31	r = 0.09	r = 0.09
Right Amygdala Volume	r = 0.31	r = 0.02	r = 0.2
Left Lateral Orbitofrontal Thickness	r = 0.31	r = 0.24	r = −0.04
Right Pars Orbitalis Thickness	r = 0.3	r = 0.06	r = 0.03
Left Isthmus Cingulate Thickness	r = 0.3	**r = −0.17**	r = 0.11
Left Paracentral Thickness	r = 0.3	r = 0.12	r = 0.29
Left Caudal Anterior Cingulate Thickness	r = 0.29	r = 0.27	r = 0.22
Left Caudate Volume	r = 0.28	r = −0.01	**r = −0.17**
Left Hippocampus Volume	r = 0.28	r = −0.05	r = 0.17
Right Bankssts Thickness	r = 0.28	r = 0.11	r = 0.28
Right Cerebellum Cortex Volume	r = 0.27	r = 0.13	r = 0.01
Left Pars Orbitalis Thickness	r = 0.27	**r = −0.28**	r = 0.17
Right Hippocampus Volume	r = 0.27	**r = −0.12**	r = 0.27
Left Cerebellum Cortex Volume	r = 0.25	r = 0.11	r = −0.02
Left Parahippocampal Thickness	r = 0.24	r = −0.05	r = 0.26
Left Medial Orbitofrontal Thickness	r = 0.24	r = 0.09	r = −0.02
Right Inferior Temporal Thickness	r = 0.24	r = −0.1	r = 0.06
Left Inferior Temporal Thickness	r = 0.21	r = −0.05	r = 0.03
Left Frontal Pole Thickness	r = 0.21	r = 0.02	r = 0.36
Right Entorhinal Thickness	r = 0.2	r = 0.12	**r = −0.1**
Left Lateral Occipital Thickness	r = 0.19	r = −0.01	r = 0.35
Right Frontal Pole Thickness	r = 0.19	**r = 0.53**	r = −0.04
Left Transverse Temporal Thickness	r = 0.18	r = 0.07	r = 0.07
Right Parahippocampal Thickness	r = 0.15	r = 0.04	r = 0.08
Right Caudate Volume	r = 0.15	r = −0.03	**r = −0.21**
Right Temporal Pole Thickness	r = 0.15	r = −0.03	r = 0.16
Left Fusiform Thickness	r = 0.13	r = 0.01	r = 0.02
Right Cuneus Thickness	r = 0.12	r = 0.03	r = 0.11

Right Transverse Temporal Thickness	r = 0.12	r = −0.08	r = 0.1
Right Isthmus Cingulate Thickness	r = 0.11	r = −0.05	r = 0.22
Right Lateral Occipital Thickness	r = 0.1	**r = −0.13**	r = 0.19
Right Caudal Anterior Cingulate Thickness	r = 0.08	**r = 0.34**	**r = −0.18**
Left Pericalcarine Thickness	r = 0.07	r = 0.13	r = −0.03
Left Entorhinal Thickness	r = 0.04	**r = −0.13**	r = 0.15
Left Temporal Pole Thickness	r = 0.04	r = 0.06	r = 0
Right Fusiform Thickness	r = 0.01	r = −0.1	r = 0
Right Lingual Thickness	r = −0.03	**r = −0.34**	r = −0.07
Left Cuneus Thickness	r = −0.04	r = 0.03	r = 0.01
Right Pericalcarine Thickness	r = −0.04	r = 0.16	r = −0.04
Left Lingual Thickness	r = −0.08	r = 0.03	**r = −0.28**
Right Medial Orbitofrontal Thickness	r = −0.13	r = 0.16	**r = −0.47**
Right Lateral Ventricle Volume	r = −0.58	r = −0.08	**r = −0.37**
Left Lateral Ventricle Volume	r = −0.6	r = −0.04	**r = −0.48**

Top contributions shown in the main text are highlighted in bold.

Supplementary Table 3. Canonical weights of the non-imaging variables to the fMRI variate.

Non-imaging Variable	All participants: sCCA Weights	Younger participants only: sCCA Weights	Older participants only: sCCA Weights
**2_back_ACC**	**r = 0.68**	r = 0.08	**r = 0.5**
**Perceived stress** (emo_psff_t)	**r = 0.68**	**r = 0.42**	r = 0.48
**Matrix_Reasoning**	**r = 0.56**	r = −0.05	**r = 0.59**
**Block_Design**	**r = 0.56**	r = 0.04	r = 0.26
**Anger-Hostility** (emo_ahff_t)	**r = 0.55**	r = 0.23	r = 0.43
**Loneliness** (emo_lff_t)	**r = 0.49**	**r = 0.34**	**r = 0.48**
**Perceived hostility** (emo_phff_t)	**r = 0.46**	r = 0.23	r = 0.3
**Sadness** (emo_sff_t)	**r = 0.45**	r = 0.26	r = 0.23
**Perceived rejection** (emo_prff_t)	**r = 0.43**	r = 0.19	**r = 0.69**
**Fear-Somatic arousal** (emo_fsaff_t)	**r = 0.42**	r = 0.26	r = 0.18
**Anger-Affect** (emo_aff_t)	r = 0.4	**r = 0.31**	r = 0.25
**Fear-Affect** (emo_faff_t)	r = 0.4	r = 0.26	r = 0.22
**Physical activity**	r = 0.39	r = −0.14	r = 0.17
**0_back_ACC**	r = 0.39	r = 0.004	r = −0.02
**SEM_Scramble_ACC**	r = 0.36	r = −0.16	r = 0.17
**Oral reading recognition** (cog_orr_t)	r = 0.32	**r = 0.4**	r = 0.002
**Picture sorting memory** (cog_psm_t)	r = 0.31	r = 0.11	r = 0.26
**Testosterone**	r = 0.3	r = 0.25	r = 0.02
**Apathy** (emo_aff_t)	r = 0.3	r = 0.15	r = 0.37
**Anger-Physical aggression** (emo_apaff_t)	r = 0.28	r = 0.22	r = 0.14
**Semantic fluency** (animal_name)	r = 0.25	r = 0.15	r = −0.32
**Estradiol**	r = 0.24	**r = 0.46**	r = 0.09
**Crystalized intelligence** (cog_cc_t)	r = 0.23	r = 0.18	r = 0.12
**FSIQ**	r = 0.2	r = −0.06	r = 0.36
**Vocabulary**	r = 0.19	r = 0.13	r = 0.24
**Pattern comparison** (cog_pcps_t)	r = 0.18	r = 0.23	r = 0.22

**Fluid intelligence** (cog_fc_t)	r = 0.14	r = 0.09	r = −0.06
**Similarities**	r = 0.13	r = −0.01	r = −0.01
**MMSE_Total**	r = 0.12	r = 0.14	r = −0.13
**Verbal fluency** (total_FAS)	r = 0.11	r = 0.01	r = −0.31
**Number of alcoholic drinks**	r = 0.09	r = 0.1	r = −0.01
**Weekend night sleep**	r = 0.09	**r = −0.31**	r = 0.04
**Sex**	r = 0.07	r = 0.07	r = −0.07
**Flanker inhibitory control and attention** (cog_fica_t)	r = 0.06	r = 0.26	r = −0.02
**Weeknight sleep**	r = 0.05	r = −0.22	r = 0.25
**Picture vocabulary** (cog_pv_t)	r = 0.01	r = −0.07	r = 0.21
**SEM_Scramble_MeanRT**	r = −0.02	r = 0.17	r = 0.31
**SEM_Scene_ACC**	r = −0.03	r = −0.02	r = 0.07
**Dimensional card change sort** (cog_dccs_t)	r = −0.04	r = −0.15	r = −0.25
**Friendship** (emo_fff_t)	r = −0.06	r = −0.28	**r = −0.42**
**High blood pressure**	r = −0.14	r = 0.12	r = −0.01
**SEM_Scene_MeanRT**	r = −0.24	r = 0.01	r = 0.14
**List sorting working memory** (cog_lswm_t)	r = −0.26	r = −0.2	**r = −0.59**
**2_back_MeanRT**	r = −0.3	r = 0.19	r = 0.26
**BMI**	r = −0.31	r = −0.12	r = −0.11
**Emotional support** (emo_esff_t)	r = −0.33	r = −0.11	**r = −0.39**
**Domain life satisfaction** (emo_dslsff_raw)	r = −0.33	r = −0.04	**r = −0.49**
**Positive affect** (emo_paff_t)	r = −0.34	**r = −0.41**	r = −0.34
**HBA1C**	r = −0.35	r = 0.0008	r = −0.16
**EAG**	**r = −0.35**	r = 0.00001	r = −0.17
**Self-efficacy** (emo_secat_t)	**r = −0.4**	r = −0.3	r = −0.2
**Instrumental support** (emo_isff_t)	**r = −0.42**	r = −0.15	r = −0.39
**Social satisfaction** (emo_sss_t)	**r = −0.45**	r = −0.29	**r = −0.63**
**Meaning and Purpose** (emo_mpff_t)	**r = −0.46**	**r = −0.4**	r = −0.38
**0_back_MeanRT**	**r = −0.46**	r = 0.05	r = 0.02
**General life satisfaction** (emo_glsff_t)	**r = −0.47**	r = −0.07	r = −0.38
**Pegboard_LH**	**r = −0.5**	**r = −0.37**	r = −0.2
**Pegboard_RH**	**r = −0.6**	r = −0.06	r = −0.33
**Age**	**r = −0.89**	**r = −0.5**	r = −0.3

Variables are described in Supplementary Table 5. Top contributions shown in the main text are highlighted in bold.

Supplementary Table 4. Demographic and cognitive information for each study group.

Variables	Younger Participants (n = 27)	Older Participants (n = 26)	P-value
**Sex (Females, n [%])**	14 [51.9%]	17 [65%]	0.5160
**Age (years)**	25.54 (3.42)	61.85 (4.37)	0.0000
**Body Mass Index (BMI)**	24.34 (3.67)	26.63 (3.79)	0.0300
**HBA1C**	5.26 (0.28)	5.48 (0.33)	0.0130
**EAG**	104.20 (8.00)	110.5 (9.36)	0.0130
**Estradiol**	1.42 (0.91)	1.18 (0.7)	0.2810
**Testosterone**	126.477 (87.55)	84.16 (55.65)	0.0420

**High Blood Pressure (n [%])**	2 [7.4%]	6 [23%]	0.2040
**Number of Alcoholic Drinks**	4.07 (1.73)	3.65 (2.56)	0.4860
**Physical Activity**	4.33 (0.76)	3.48 (1.2)	0.0030
**Weeknight Sleep**	7.96 (1.15)	7.9 (0.86)	0.8330
**Weekend Night Sleep**	8.43 (1.22)	8.09 (1.03)	0.2800
**MMSE_total**	28.96 (1.43)	28.46 (1.77)	0.2610
**Block_design**	53.89 (10.17)	41.31 (8.24)	0.0000
**Vocabulary**	41.63 (3.66)	40.81 (3.52)	0.4090
**Matrix_reasoning**	24.11 (2.15)	21.54 (3.1)	0.0010
**Similarities**	35.93 (3.63)	34.85 (3.44)	0.2710
**FSIQ**	114.15 (9.31)	111.92 (7.63)	0.3470
**Verbal fluency (Total_FAS)**	47.96 (11.45)	43.23 (10)	0.1160
**Semantic fluency (Animal_name)**	24.41 (3.78)	21.15 (4.52)	0.0060
**(cog_orr_t)**	54.85 (7.96)	51.12 (4.38)	0.4000
**cog_pv_t**	52.67 (8.51)	53 (5.43)	0.8660
**cog_lswm_t**	51.70 (8.82)	52.85 (8.08)	0.6250
**cog_pcps_t**	59.33 (12.74)	57.08 (13.32)	0.5310
**cog_psm_t**	57.78 (12.98)	51.23 (12.57)	0.0680
**cog_fica_t**	47.33 (11.28)	46.88 (7.5)	0.8660
**cog_dccs_t**	57.11 (10.39)	55.88 (9.24)	0.6520
**cog_fc_t**	57.04 (9.98)	53.88 (9.8)	0.2510
**cog_cc_t**	55.19 (9.32)	52.27 (4.42)	0.1540
**pegboard_rh**	18.40 (1.93)	21.74 (2.94)	0.0000
**pegboard_lh**	19.75 (1.74)	22.31 (3.21)	0.0010
**emo_ahff_t**	57.67 (8.10)	48.81 (9.43)	0.0010
**emo_aaff_t**	55.74 (8.07)	49.92 (9.16)	0.0170
**emo_apaff_t**	52.22 (11.19)	47.73 (7.45)	0.0930
**emo_phff_t**	53.81 (8.32)	47.42 (6.26)	0.0030
**emo_lff_t**	58.07 (10.16)	50.54 (11.07)	0.0130
**emo_prff_t**	52.63 (9.03)	48.62 (9.22)	0.1160
**emo_secat_t**	47.89 (6.38)	53.38 (8.86)	0.0120
**emo_psff_t**	55.85 (8.43)	43.42 (9.54)	0.0000
**emo_faff_t**	52.67 (10.38)	45.88 (8.74)	0.0130
**emo_fsaff_t**	50.41 (8.54)	44.23 (6.54)	0.0050
**emo_glsff_t**	50.22 (5.92)	56.92 (9.85)	0.0040
**emo_dslsff_raw**	50.59 (8.11)	53.96 (7.38)	0.1200
**emo_mpff_t**	47.19 (9.45)	53.77 (10.15)	0.0180
**emo_paff_t**	48.15 (8.71)	51.62 (8.80)	0.1560
**emo_sff_t**	52.78 (10.69)	44.19 (9.14)	0.0030
**emo_aff_t**	46.78 (8.50)	43.73 (6.16)	0.1420
**emo_esff_t**	47.48 (9.89)	51.77 (7.60)	0.0840
**emo_fff_t**	49.67 (10.42)	47.04 (10.49)	0.3650
**emo_isff_t**	47.63 (8.80)	53.96 (9.89)	0.0170
**emo_sss_t**	45.74 (10.08)	50.85 (9.57)	0.0640
**SEM_Scene_MeanRT (ms)**	920.37 (115.09)	1007.11 (141.53)	0.0180
**SEM_Scene_ACC**	98% (2%)	98% (3%)	0.5780
**SEM_Scramble_MeanRT (ms)**	1741.41 (221.36)	1823.94 (267.77)	0.2270
**SEM_Scramble_ACC**	92% (5%)	85% (11%)	0.0120

**0_back_MeanRT (ms)**	799.80 (137.06)	1106.51 (320.97)	0.0000
**2_back_MeanRT (ms)**	1050.99 (182.55)	1250.59 (217.79)	0.0003
**0_back_ACC**	94% (10%)	81% (16%)	0.0010
**2_back_ACC**	95% (5%)	81% (12%)	0.0000

All continuous variables are shown as mean (std). All variables are described in Supplementary Table 5. *P*-values reflect group differences from either a *t*-test or a chi-squared test as appropriate.

Supplementary Table 5. Description of the 59 behavioral variables.

Variable Name	Variable Description
Sex	Male and Female
Age	Age in years
BMI	Current Body Mass Index
HbA1c	Hemoglobin A1C
EAG	Estimated average glucose measured (mg/dl)
Estradiol	Mean of two results of the same sample (pg/ml)
Testosterone	Mean of two results of the same sample (pg/ml)
High Blood Pressure	Personal history of high blood pressure. Yes or No
Number of Alcoholic Drinks	Average number of alcoholic drinks consumed/month. Computed to Likert scale score (0–8, with 0: no drinks, and 8: daily drinks)
Physical Activity	Composite score reflecting the degree of intensity and frequency of exercise activity. Computed scale score ranging (0–6, with 0: no activity, 6: daily exercise with high intensity).
Weeknight Sleep	Average hours of sleep on a weeknight
Weekend Night Sleep	Average hours of sleep on a weekend night
MMSE_total	Total score for the Mini-Mental Status Exam
Block_design	Raw score for the block design section on the WASI-II. Participants are asked to re-create the block pattern they are shown as quickly as they are able.
Vocabulary	Raw score for the vocabulary section on the WASI-II. Participants are asked to define the meaning of words they are given.
Matrix_reasoning	Raw score for the matrix reasoning section on the WASI-II. Participants are asked to identifying the design to complete the pattern shown.
Similarities	Raw score for the similarities section on the WASI-II. Participants are asked to identify the similarity between two words.
FSIQ	Full Scale IQ score taken from sum of T-Scores on the WASI-II combining the block design, vocabulary, matrix reasoning, and similarities sections
Total_FAS	**Verbal Fluency**. Sum of total number of words participant verbalizes starting with a F, A and S in one minute each.
Animal_name	**Semantic Fluency**. Sum of total number of animals participant verbalizes in one minute.
cog_orr_t	**Oral reading recognition** T-Score. Participant is asked to read and pronounce letters and words as accurately as possible. Completed via NIH Toolbox.
cog_pv_t	**Picture Vocabulary** T-Score. Participant selects the picture (out of four pictures presented) that most closely matches the meaning of the word. Completed via NIH Toolbox.
cog_lswm_t	**List Sorting Working Memory** T-Score. Participant is asked to verbally repeat back objects that were shown with an auditory explanation, but re-order them by size from smallest to largest. The list of objects continually becomes longer. Completed via NIH Toolbox.
cog_pcps_t	**Pattern Comparison Processing Speed** T-Score. Participant is asked to indicate whether two pictures are matching or not. Items are simple so as to purely measure processing speed. They are asked to respond as quickly and accurately as they are able to. Completed via NIH Toolbox.

cog_psm_t	**Picture Sorting Memory** T-Score. Participant views brief action activities presented as pictures with auditory explanation on a screen and attempt to place the pictures back in the same order they were shown on the screen. Completed via NIH Toolbox
cog_fica_t	**Flanker Inhibitory Control and Attention** T-Score. Participant must indicate which direction the middle arrow is pointing in a row of five arrows as quickly and accurately as they are able.
cog_dccs_t	**Dimensional Card Change Sort** T-Score. Respondents are asked to indicate either the matching color or shape of a target picture based upon the prompt they were given as quickly and accurately as they are able. Completed via NIH Toolbox.
cog_fc_t	**The fluid (intelligence) composite** T score for the NIH Toolbox including the dimensional card change sort, flanker inhibitory, picture sequence memory, list sorting, and pattern comparison measures.
cog_cc_t	**The crystalized (intelligence) composite score for the NIH Toolbox** including picture vocabulary and oral reading recognition.
pegboard_RH	Participants are asked to place pegs, with their right hand, in holes one at a time as quickly as they are able and then take them out of the holes one at a time as they are able. Total time (sec). Completed with a physical peg board and the NIH Toolbox.
pegboard_LH	Participants are asked to place pegs, with their left hand, in holes one at a time as quickly as they are able and then take them out of the holes one at a time as they are able. Total time (sec). Completed with a physical peg board and the NIH Toolbox.
emo_ahff_t	**Anger and hostility questions.** Computed T-Score. Completed via NIH Toolbox.
emo_aaff_t	**Anger with affect questions.** Computed T-Score. Completed via NIH Toolbox.
emo_apaff_t	**Anger with physical aggression questions.** Computed T-Score. Completed via NIH Toolbox.
emo_phff_t	**Perceived hostility questions.** Computed T-Score. Completed via NIH Toolbox.
emo_lff_t	**Loneliness questions.** Computed T-Score. Completed via NIH Toolbox.
emo_prff_t	**Perceived rejection questions.** Computed T-Score. Completed via NIH Toolbox.
emo_secat_t	**Self-efficacy questions.** Computed T-Score. Completed via NIH Toolbox.
emo_psff_t	**Perceived stress questions.** Computed T-Score. Completed via NIH Toolbox.
emo_faff_t	**Fear with affect questions.** Computed T-Score. Completed via NIH Toolbox.
emo_fsaff_t	**Fear with somatic arousal questions.** Computed T-Score. Completed via NIH Toolbox.
emo_glsff_t	**General life satisfaction questions.** Computed T-Score. Completed via NIH Toolbox.
emo_dslsff_raw	**Domain-specific life satisfaction questions.** Computed raw score. Completed via NIH Toolbox.
emo_mpff_t	**Meaning and purpose questions.** Computed T-Score. Completed via NIH Toolbox.
emo_paff_t	**Positive affect questions.** Computed T-Score. Completed via NIH Toolbox.
emo_sff_t	**Sadness questions.** Computed T-Score. Completed via NIH Toolbox.
emo_aff_t	**Apathy questions.** Computed T-Score. Completed via NIH Toolbox.
emo_esff_t	**Emotional support questions.** Computed T-Score. Completed via NIH Toolbox.
emo_fff_t	**Friendship questions. Computed** T-Score. Completed via NIH Toolbox.
emo_isff_t	**Instrumental support questions.** Computed T-Score. Completed via NIH Toolbox.
emo_sss_t	**Social satisfaction summary** T-Score. Completed via NIH Toolbox.

SEM_Scene_MeanRT	During the SEM fMRI task, mean reaction time (in ms) for accurate responses only during the scene condition (participant is asked to determine if the picture is an indoor or outdoor setting).
SEM_Scene_ACC	During the SEM fMRI task, total accuracy (in %) for the scene condition.
SEM_Scramble_MeanRT	During the SEM fMRI task, mean reaction time (in ms) for accurate responses only during the control condition (participant is asked to determine if the scrambled picture has 2 identical halves).
SEM_Scramble_ACC	During the SEM fMRI task, total accuracy (in %) for the control condition.
0_back_MeanRT	During the n-back fMRI task, mean reaction time (in ms) for accurate responses only during the 0-back condition.
2_back_MeanRT	During the n-back fMRI task, mean reaction time (in ms) for accurate responses only during the 2-back condition.
0_back_ACC	During the n-back fMRI task, total accuracy (in %) for the 0-back condition.
2_back_ACC	During the n-back fMRI task, total accuracy (in %) for the 2-back condition.

Supplementary Table 6. Activation peak for the N-Back task.

Region	T	x	y	z
**Left Inferior Parietal Cortex**	6.81	−38	−50	46
**Right Inferior Parietal Cortex**	6.78	38	−46	46
**Left Middle Frontal Cortex**	6.26	−22	6	54
**Left Inferior Parietal Cortex**	6.18	−50	−44	46
**Right Middle Frontal Cortex**	7.84	28	8	52
**Left SMA**	6.42	−6	12	50
**Right Cerebellum**	7.58	30	−58	−34
**Right Precuneus**	7.30	10	−60	56
**Left Cerebellum**	7.06	−42	−62	−36
**Left Middle Frontal Cortex**	5.63	−30	44	16
**Right Thalamus**	5.22	14	−8	10
**Left Insula**	4.92	−32	16	2

MNI Coordinates. Abbreviation: SMA: Supplementary Motor Area. Activation peaks are shown on Supplementary Figure 2B.

Supplementary Table 7. Activation peak for the scene encoding memory task.

Region	T	x	y	z
**Right Fusiform Gyrus**	18.01	26	−46	−10
**Left Fusiform Gyrus**	17.22	−20	−36	−16
**Right Lingual Gyrus**	18.48	6	−76	−4
**Right Retrosplenial Cortex**	18.42	10	−52	8
**Left Retrosplenial Cortex**	18.18	−10	−56	10
**Left Orbital MPFC**	13.36	−6	20	−14
**Left Middle Occipital Cortex**	12.70	−46	−72	26
**Right Middle Occipital Cortex**	11.76	48	−76	2
**Right Middle Temporal Cortex**	9.73	56	−2	−22

MNI Coordinates. Abbreviation: MPFC: Medial Prefrontal Cortex.

Supplementary Table 8. Activation peak for the verb generation task.

Region	T	x	y	z
**Right Cerebellum**	18.25	30	−60	−30
**Left SMA**	16.02	−8	4	64
**Left Inferior Frontal Gyrus**	15.27	−48	12	6
**Left Cerebellum**	13.07	−42	−64	−28
**Right anterior Insula**	11.94	42	16	−4
**Left Inferior Parietal**	10.10	−30	−62	42
**Left Hippocampus**	10.09	−32	−18	−10
**Right Putamen**	10.00	20	2	10

MNI Coordinates. Abbreviation: SMA: Supplementary Motor Area.
